# LDNFSGB: prediction of long non-coding rna and disease association using network feature similarity and gradient boosting

**DOI:** 10.1186/s12859-020-03721-0

**Published:** 2020-09-03

**Authors:** Yuan Zhang, Fei Ye, Dapeng Xiong, Xieping Gao

**Affiliations:** 1 School of Mathematics and Computational Science, Xiangtan University, Xiangtan 411105, China; 2 Key Laboratory of Intelligent Computing and Information Processing of Ministry of Education, Xiangtan University, Xiangtan 411105, China; 3 Department of Computational Biology, Ithaca, New York 14853, USA; 4 Weill Institute for Cell and Molecular Biology, Cornell University, Ithaca, New York 14853, USA; 5 College of Medical Imaging and Inspection, Xiangnan University, Chenzhou 423000, China

**Keywords:** lncRNA-disease association, Prediction, Network feature similarity, Gradient boosting

## Abstract

**Background:**

A large number of experimental studies show that the mutation and regulation of long non-coding RNAs (lncRNAs) are associated with various human diseases. Accurate prediction of lncRNA-disease associations can provide a new perspective for the diagnosis and treatment of diseases. The main function of many lncRNAs is still unclear and using traditional experiments to detect lncRNA-disease associations is time-consuming.

**Results:**

In this paper, we develop a novel and effective method for the prediction of lncRNA-disease associations using network feature similarity and gradient boosting (LDNFSGB). In LDNFSGB, we first construct a comprehensive feature vector to effectively extract the global and local information of lncRNAs and diseases through considering the disease semantic similarity (DISSS), the lncRNA function similarity (LNCFS), the lncRNA Gaussian interaction profile kernel similarity (LNCGS), the disease Gaussian interaction profile kernel similarity (DISGS), and the lncRNA-disease interaction (LNCDIS). Particularly, two methods are used to calculate the DISSS (LNCFS) for considering the local and global information of disease semantics (lncRNA functions) respectively. An autoencoder is then used to reduce the dimensionality of the feature vector to obtain the optimal feature parameter from the original feature set. Furthermore, we employ the gradient boosting algorithm to obtain the lncRNA-disease association prediction.

**Conclusions:**

In this study, hold-out, leave-one-out cross-validation, and ten-fold cross-validation methods are implemented on three publicly available datasets to evaluate the performance of LDNFSGB. Extensive experiments show that LDNFSGB dramatically outperforms other state-of-the-art methods. The case studies on six diseases, including cancers and non-cancers, further demonstrate the effectiveness of our method in real-world applications.

## Background

Cumulative evidence shows that only ∼2 percent of protein-coding genes are in the human genome and the remaining ∼98 percent of the human genome are classified as non-coding RNAs (ncRNAs) [1]. Many studies in recent years suggest that the interaction of ncRNA and protein has a positive effect on many biological processes, such as protein synthesis, gene expression, RNA processing, and development regulation [2]. Based on the expression and function, ncRNAs are divided into ribosomal RNA, transfer RNA, small nuclear RNA, and small nucleoli RNA [3]. According to its size, regulatory ncRNAs can be further classified as small ncRNA (∼18-31nt, such as miRNA, siRNA, and piRNA), medium ncRNA (∼31-200nt) and long ncRNA (from 200nt up to several hundred kb, such as lincRNA and microRNA) [4].

Long non-coding RNAs (lncRNAs) play an increasingly important role in some fundamental biological processes such as translational regulation, cell cycle regulation, epigenetic regulation, splicing, differentiation, and immune response [5]. For example, GAS5 inhibits cell invasion and tumor growth [6]. HOTAIR, a 2.2 kb gene in the HOXC locus, plays a key role in epigenetic regulation in cancer [7]. Especially, many studies demonstrate that mutations and disorders of lncRNAs are associated with human complex diseases, including Alzheimer’s disease (AD), glioma, breast cancer, psychiatric disease, cardiovascular disease, AIDS, and glaucoma [8]. For example, the synthesis of 51A can promote the expression of alternative splicing SORL1 variants. Quantitative RT-PCR is often used to verify the overexpression in the metastatic samples. Nevertheless, the metastasis of NSCLC patients is significantly related to MALAT-1 [9]. Forced expression of HOTAIR in epithelial cancer cells induces genome-wide Polycomb repression complex 2 (PRC2) to retarget to a more similar pattern of embryonic fibroblasts, leading to gene expression changes, and increase cancer invasion and metastasis. In contrast, the loss of HOTAIR can inhibit cancer invasion, especially in cells with excessive PRC2 activity. These findings suggest that lncRNAs have a positive role in regulating the epigenome of cancer and may be an important target for cancer diagnosis and treatment [10]. Therefore, predicting the potential association between diseases and lncRNAs can not only promote the understanding of molecular mechanisms for human diseases at the level of lncRNAs, but also better identify biomarkers for the diagnosis, treatment, prognosis, and prevention of human diseases [11, 12] However, it is costly and time-consuming for traditional biological experiments in discovering potential lncRNA-disease associations [13]. Besides, classical biological experiment methods are usually not made available for the analysis of a large number of candidates [14]. Therefore, it is essential to propose an effective and efficient computational model for predicting lncRNA-disease associations [12, 15].

In the past decades, various computation models based on different mathematical theories have been proposed to address this issue [16, 17]. These methods can be divided into two categories, i.e., network-based methods and machine learning-based methods. The network-based methods mainly use biological information related to lncRNAs for the prediction. Chen et al. [11] proposed the Laplacian regularized least squares model (LRLSLDA) to predict the lncRNA-disease association, which is the first computational model used to predict lncRNA-disease association. Zhou et al. [18] proposed RWRHLD as a candidate for the lncRNA-disease association by integrating miRNA-related lncRNA-lncRNA crosstalk network, disease-disease similarity network, and known lncRNA-disease association network. Chen et al. [19] introduced KATZLDA to predict the lncRNA-disease association.

In [20], a hypergeometric distribution model for lncRNA-disease association inference was developed to predict lncRNA-disease association by integrating miRNA-disease association and lncRNA-miRNA interaction. Ping et al. [21] constructed a two-part heterogeneous network obeying the power-law distribution based on known lncRNA-disease correlations to predict potential lncRNA-disease association sample pairs. The implementation of this method requires the assumption that lncRNAs related to the same or similar diseases may have similar functions [22]. Chen et al. [23] proposed ILDMSF to identify an association between lncRNAs and diseases by using multi-similarity fusion strategy. This method solves the problem of unsatisfactory prediction results using a single similarity measure or a linear method that fuses different similarities. Yang et al. [9] introduced genetic information to identify lncRNA-related diseases. They constructed a coding-non-coding gene-disease bipartite network based on known associations diseases and disease-causing genes. Lu et al. [24] developed a method named SIMCLDA by using inductive matrix completion to discover the potential lncRNA-disease association. What is common to all of these approaches is the assumption that molecules with similar structures or ligands have similar functions. However, molecules with similar structures or ligands may not have similar functions. Besides, the performance of the matrix fusion method may decrease when the known association information is insufficient. Therefore, these methods do not reveal the inherent logical association between lncRNAs and complex diseases.

For the machine learning-based methods, some classical algorithms are often used to predict the potential association between lncRNAs and diseases. Yu et al. [25] firstly constructed an updated tripartite network by integrating the miRNA-disease interaction network, miRNA-lncRNA interaction network, and lncRNA-disease network, and then predicted lncRNA-disease association based on Naïve Bayesian classifier and collaborative filtering model. In [26], InfDisSim was proposed to predict disease-related ncRNA based on a damped random walk model. Sun et al. [27] introduced RWRlncD to infer the lncRNA-disease association by implementing a restart random walk method on the lncRNA functional similarity network. Li et al. [28] also proposed a local random walk model (LREWHLDA) to predict the lncRNA-disease association. Yao et al. [29] proposed to predict lncRNA-disease associations based on random forests. In [30], a clustering algorithm was proposed based on unsupervised learning to predict the lncRNA-disease association. Wang et al. [31] established a prediction model called Internal Random Walk with Restart (IIRWR) to predict lncRNA-related diseases. Lan et al. [32] introduced LDAP to identify potential associations between lncRNAs and diseases by using a bagging support vector machine (SVM) classifier. Although these methods have achieved varying degrees of success, they did not comprehensively take into account the global information between lncRNAs and diseases, internal information between lncRNAs, internal information between diseases, and the sparsity of known lncRNA-disease association, which are all thought to be able to contribute to the prediction.

Recently, Xiao et al. [33] proposed BPLLDA to predict lncRNA-disease associations. This method mainly predicted the degree of association between lncRNAs and diseases by calculating the paths connecting them and their lengths. Although this method improved the prediction accuracy, the semantic similarity calculation in this method only simply considered the local information of the semantics. Actually, global semantic information on the disease is also important because the frequency of the disease may affect its contribution. Therefore, it is necessary to consider the features of disease and lncRNA more comprehensively to accurately predict the associations between lncRNAs and diseases.

In this paper, we propose a novel method, called LDNFSGB, for the large-scale lncRNA-disease association prediction. Firstly, we construct a comprehensive feature vector to effectively extract the global and local information of diseases and lncRNAs using a disease similarity (DISS) heterogeneous network and a lncRNA similarity (LNCS) heterogeneous network. Specifically, DISS is constructed by combining the disease Gaussian interaction profile kernel similarity (DISGS) and the disease semantic similarity (DISSS) heterogeneous network. And LNCS is constructed by integrating the lncRNA Gaussian interaction profile kernel similarity (LNCGS) and lncRNA function similarity (LNCFS) heterogeneous network. Here, for the calculation of either DISSS or LNCFS, the average derived from two strategies is taken as the final score. In particular, DISSS1 (LNCFS1) is used to consider the local information of disease semantics (lncRNA functions) and DISSS2 (LNCFS2) is for the global information of disease semantics (lncRNA functions). Secondly, an autoencoder is used to reduce the dimensionality of the feature vector to get the optimal feature parameter from the original feature set. Furthermore, considering the distribution characteristics of the data, we employ a gradient boosting algorithm to predict the lncRNA-disease associations. Finally, three validation methods, including the hold-out, leave-one-out cross-validation (LOOCV), and ten-fold cross-validation (10-fold CV), are implemented to demonstrate the prediction performance of the proposed LDNFSGB on three publicly available datasets, i.e., LncRNADisease, Lnc2Cancer, and LncRNADisease2.0. The experimental results indicate that the proposed LDNFSGB achieves 0.9761, 0.9447, 0.9933 in terms of AUC values using hold-out on the three datasets respectively, which outperforms the state-of-the-art methods for predicting candidate disease lncRNAs. Additional case studies on six diseases, including colorectal cancer, osteosarcoma, cervical cancer, glioma, heart failure, and AD, further show that LDNFSGB can successfully predict potential disease-related lncRNAs candidates.

## Results

To verify the performance of the proposed LDNFSGB, a series of experiments are conducted. (1) In order to construct the best similarity features, we implement a comparative experiment on the LncRNADisease dataset based on different features and compare and analyze the experimental results of LDNFSGB under different feature vectors. (2) To verify the performance of the gradient boosting algorithm, we conduct an comparison experiment on LncRNADisease using eight different classifiers including Gradient Boosting, SVM, Naïve Bayes, Logistic Regression, Random Forest, Rotation Forest, AdaBoost, and Deep Extreme Learning Machine (DELM). (3) We use three validation methods (i.e., hold-out, LOOCV, and 10-fold CV) to comprehensively evaluate the performance of the proposed LDNFSGB on three publicly available datasets. (4) To evaluate the overall performance of LDNFSGB, we compare the results of the proposed method with several state-of-the-art approaches in the literature.

### Validation methods

#### Hold-out

The reserved method is to divide the dataset into a training set and a test set according to a certain ratio, and then use the training set to learn the model. The test set is used for lncRNA-disease association prediction and model performance evaluation. A large number of experiments have demonstrated that the best training results can be obtained by randomly dividing the datasets according to the 8 to 2 ratio [34–37].

#### Leave-one-out cross-validation

Although hold-out can obtain better test results, the randomness of the training sample and test sample division causes a certain bias in the results. Thus, LOOCV is chosen as another validation method. For the LOOCV, traverse all the samples according to the principle of leaving one sample as the test set and all the remaining samples as the training set. Finally, we take the average of all test results as the final result. In general, LOOCV can obtain relatively stable results because of the large number of runs.

#### Ten-fold cross-validation

We herein use the 10-fold CV to further evaluate the performance of the proposed method. The basic principle of 10-fold CV is to divide all data randomly into 10 disjoint subsets. For each round, 9 subsets are used for training and the remaining for testing. After 10 rounds, the average of the 10 results is used as the final evaluation result. Overall, the 10-fold CV method saves time to some extent and reduces the deviation caused by the random partition of data.

#### Performance metric

To evaluate the performance of LDNFSGB, the receiver operating characteristic (ROC) curves are utilized and the area under ROC (AUC) values are calculated. Also, five other metrics are used for the evaluation, including Accuracy (Acc), Sensitivity (Sen), Specificity (Spe), Precision (Pre), and Matthews Correlation Coefficient (MCC), which are defined as

1 $$ Acc=\frac{TP+TN}{TP+FN+TN+FP}  $$

2 $$ Sen=\frac{TP}{TP+FN}  $$

3 $$ Spe=\frac{TN}{TN+FP}  $$

4 $$ Pre=\frac{TP}{TP+FP}  $$

5 $$ MCC \! \! = \! \! \frac {{TP \! \! \times \! TN \! \!- \! \! FP \! \! \times \! \! {\rm{FN}}}}{{\sqrt {(TP \! + \! {\rm{FN)}} \! \times \! {\rm{(}}TP \! + \! FP{\rm{)}} \! \times \! {\rm{(}}TN \! + \! {\rm{FN)}} \! \times \! {\rm{(}}TN \! + \! FP{\rm{)}}} }}  $$

where *TP*, *FP*, *TN*, *FN* are the number of true positives, false positives, true negatives, and false negatives in the confusion matrix, respectively.

### Parameter settings

In LDNFSGB, different parameters of the autoencoder and gradient boosting algorithm will generate different prediction results. The parameters settings and implementation details of our experiments are presented as following. For the autoencoder, we use the Keras library and set the batch size and epoch to 128 and 100, respectively. For gradient boosting, we set the maximum tree depth *d* to 3, the number of regression tree *q* to 1200, the random seed to 0, and the learning rate *η* to 0.1. All the experiments are implemented using Pycharm2019 on a PC (Intel i5-7500, 3.4GHz CPU, and 8-GB RAM).

### Overall performance on the LncRNADisease dataset

**Fig. 1:**
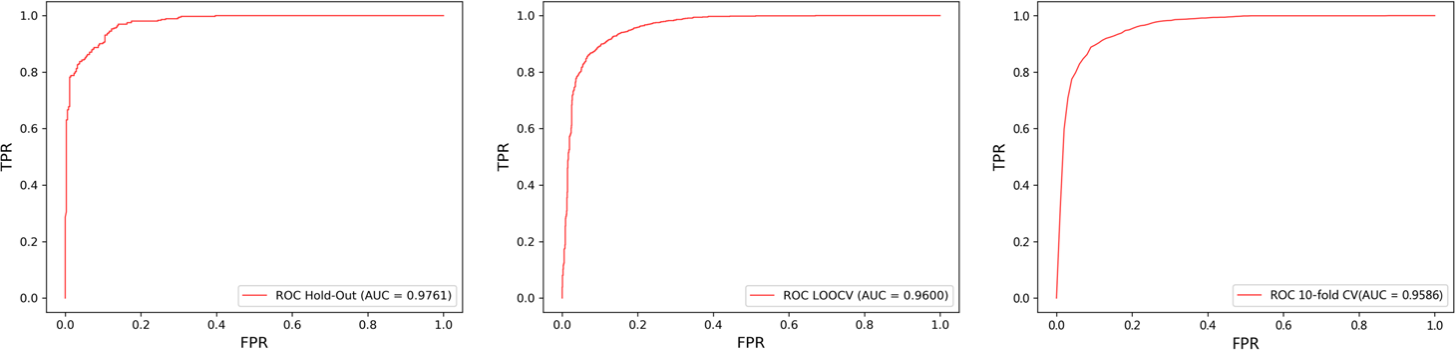
ROC curves of LDNFSGB for lncRNA-disease association prediction on LncRNADisease under hold-out, LOOCV, and 10-fold CV

Firstly, to verify the performance of our method, three validation methods including hold-out, LOOCV, and 10-fold CV are evaluated on the LncRNADisease dataset. Among them, LDNFSGB using hold-out obtains the highest result with an AUC of 0.9761. The average values of AUCs obtained by LOOCV and 10-fold CV are 0.96 and 0.9586, respectively. The ROC curves of LDNFSGB using three validation methods are shown in Fig. 1. In particular, the closer AUC is to 1, the better the predicted result is. Besides, the closer the ROC curve is to the top, the better the prediction performance is. For three validation methods, LOOCV has the most stable results with the highest computational cost. While hold-out achieves a highest accuracy, the results are a little bit unstable because of random data split. By contrast, 10-fold CV gets a good balance. Overall, the high results obtained by these three validation methods show that the proposed LDNFSGB is effective for lncRNA-disease association prediction.

### Comparison with different features

Most of the existing methods calculate the lncRNA similarity and disease similarity from a local perspective and they do not comprehensively consider the sparseness and globality of the feature matrix. In this section, we construct four tetramerous heterogeneous networks (THN1, THN2, THN3, and THN4), six tripartite heterogeneous networks (TriHN1, TriHN2, TriHN3, TriHN4, TriHN5, and TriHN6), and four duplex heterogeneous networks (DHN1, DHN2, DHN3, and DHN4) on LncRNADisease based on the disease semantic similarity, the lncRNA function similarity, the lncRNA Gaussian profile kernel similarity, the disease Gaussian profile kernel similarity, and the known disease-lncRNA interaction for comparison. Details are listed in Table 1. We construct different feature vectors based on these heterogeneous networks and take them as input features for the prediction.

**Table 1:** The detailed feature composition information of different heterogeneous networks. DISSS, LNCFS, LNCGS, DISGS, LNCDIS represent different features

Network	DISSS	LNCFS	LNCGS	DISGS	LNCDIS
FHN	*√*	*√*	*√*	*√*	*√*
THN1		*√*	*√*	*√*	*√*
THN2	*√*	*√*	*√*		*√*
THN3	*√*	*√*		*√*	*√*
THN4	*√*		*√*	*√*	*√*
TriHN1	*√*			*√*	*√*
TriHN2	*√*		*√*		*√*
TriHN3			*√*	*√*	*√*
TriHN4		*√*		*√*	*√*
TriHN5		*√*	*√*		*√*
TriHN6	*√*	*√*			*√*
DHN1	*√*				*√*
DHN2				*√*	*√*
DHN3			*√*		*√*
DHN4		*√*			*√*

**Fig. 2:**
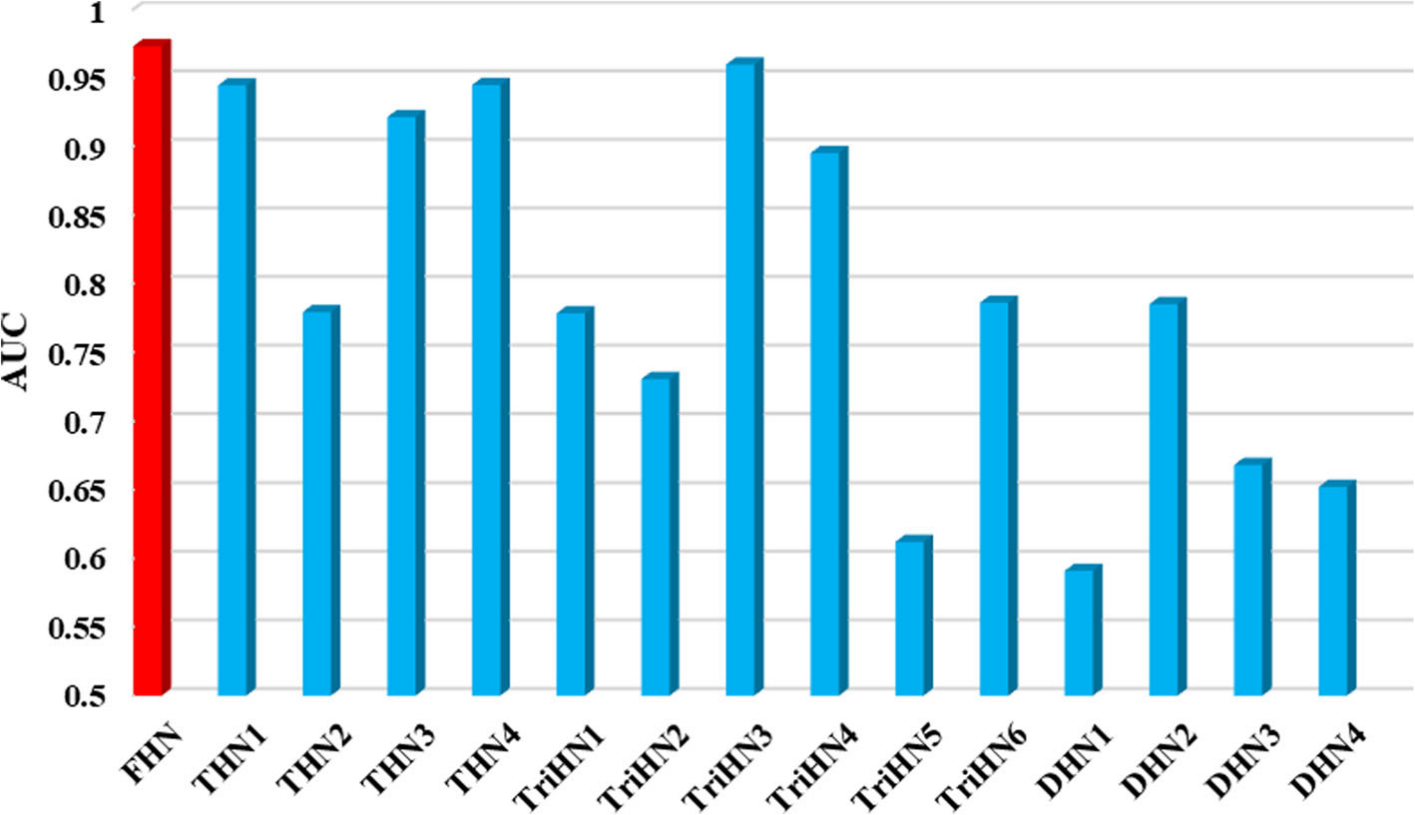
Comparison of AUC values using different features on LncRNADisease under hold-out

Comparison results on LncRNADisease are illustrated in Fig. 2. We can find that using the feature vector obtained by FHN can achieve the highest AUC with 0.9761, which is higher than other results. It is verified that the feature vector by integrating the lncRNA-disease interaction, disease semantic similarity, lncRNA functional similarity, Gaussian profile kernel similarity for lncRNAs, and Gaussian profile kernel similarity for diseases performs better than other feature vectors, is effective for the lncRNA-disease association prediction.

### Comparison with different classifiers

**Fig. 3:**
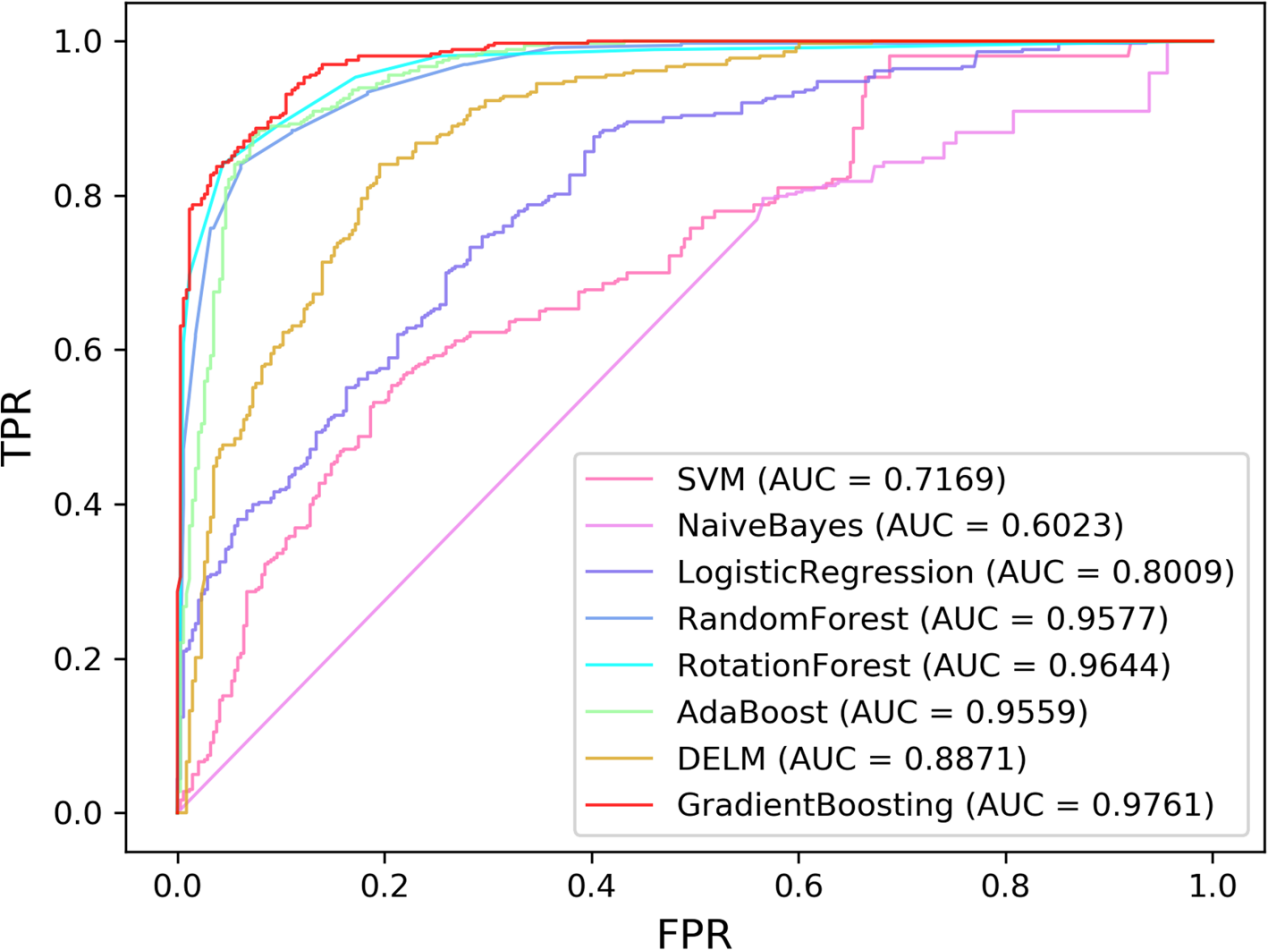
ROC curves of all classifiers for lncRNA-disease association prediction on LncRNADisease under hold-out

**Fig. 4:**
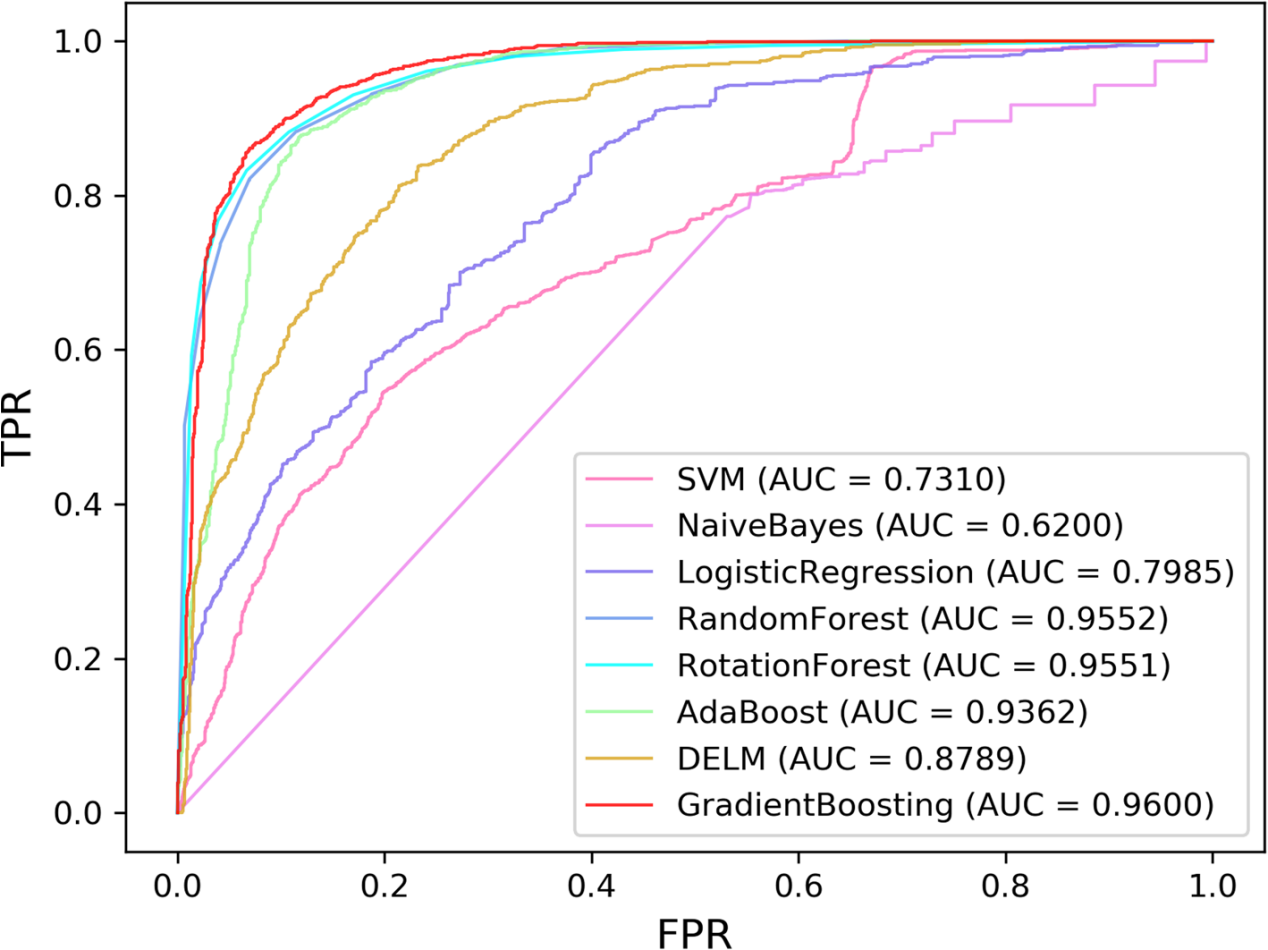
ROC curves of all classifiers for lncRNA-disease association prediction on LncRNADisease under LOOCV

**Fig. 5:**
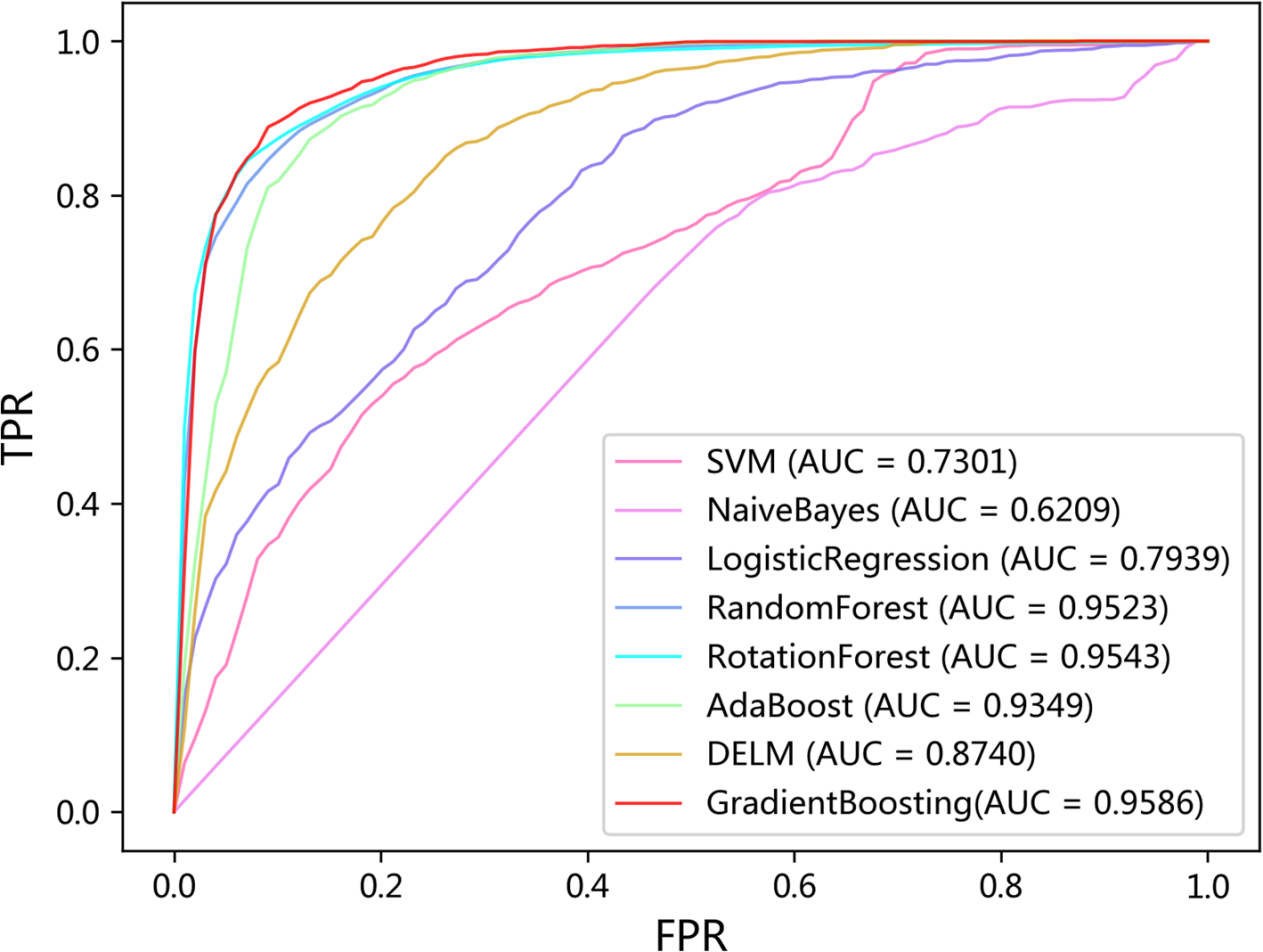
ROC curves of all classifiers for lncRNA-disease association prediction on LncRNADisease under 10-fold CV

To evaluate the performance of the gradient boosting, we also compare it with other popular classifiers. To be fair, the same data are used for all classifiers.The ROC curves of these eight classifiers using hold-out, LOOCV, and 10-fold CV on the LncRNADisease dataset are summarized in Figs. 3, 4 and 5. The comprehensive indicators by calculating confusion matric, including Acc, Sen, Spe, Pre, and MCC, which are illustrated in Tables 2, 3 and 4.

**Table 2:** Hold-out test results of LDNFSGB on LncRNADisease using different classifiers

Classifier	Acc	Sen	Spe	Pre	MCC
SVM	0.6189	0.8071	0.4198	0.5955	0.2468
Naïve Bayes	0.5736	0.8429	0.2886	0.5563	0.1585
Logistic Regression	0.7181	0.6997	0.7376	0.7383	0.4373
Random Forest	0.8852	0.8842	0.8862	0.8916	0.7704
Rotation Forest	0.9022	0.9283	0.8746	0.8868	0.8050
Ada Boosting	0.8824	0.9311	0.8309	0.8535	0.7674
DELM	0.8116	0.8787	0.7405	0.7818	0.6267
**Gradient Boosting**	**0.9138**	**0.9311**	**0.8950**	**0.9037**	**0.8273**

**Table 3:** LOOCV test results of LDNFSGB on LncRNADisease using different classifiers

Classifier	Acc	Sen	Spe	Pre	MCC
SVM	0.6252	0.8011	0.4492	0.5926	0.2675
Naïve Bayes	0.5834	0.8572	0.3099	0.5540	0.1997
Logistic Regression	0.7101	0.7053	0.7150	0.7122	0.4204
Random Forest	0.8798	0.8787	**0.8810**	**0.8807**	0.7597
Rotation Forest	0.8878	0.9087	0.8668	0.8722	0.7763
Ada Boosting	0.8719	0.9093	0.8345	0.8460	0.7459
DELM	0.7968	0.8572	0.7365	0.7649	0.5981
**Gradient Boosting**	**0.8975**	**0.9257**	**0.8657**	**0.8733**	**0.7929**

**Table 4:** 10-fold CV test results of LDNFSGB on LncRNADisease using different classifiers

Classifier	Acc	Sen	Spe	Pre	MCC
SVM	0.6218	0.8068	0.4368	0.5890	0.2625
Naïve Bayes	0.5824	0.8572	0.3076	0.5533	0.1970
Logistic Regression	0.7028	0.6940	0.7116	0.7069	0.4061
Random Forest	0.8816	0.8838	**0.8794**	**0.8813**	0.7641
Rotation Forest	0.8784	0.9127	0.8442	0.8574	0.7592
Ada Boosting	0.8677	0.9042	0.8312	0.843	0.7376
DELM	0.7932	0.8606	0.7258	0.7598	0.5925
**Gradient Boosting**	**0.8946**	**0.9240**	0.8652	**0.8736**	**0.7913**

Although the Spe and Pre values of gradient boosting are slightly lower than those of random forest, the Acc, Sen, MCC, and AUC values are the highest across the hold-out, LOOCV, and 10-fold CV. Figs. 3, 4, and 5 also show that the ROC curves of gradient boosting are located at the top of all figures. Therefore, the results verify that gradient boosting has better performance than other classifiers. We herein conclude the reasons as following: (1) The performance of SVM is sensitive to data. The choice of kernel function and the setting of parameters could also affect the final result. (2) The premise of using the Naïve Bayes is to assume that the samples are independently distributed. However, our data may not follow such an assumption. (3) Although DELM can reduce the complexity of the model, the experimental results are unstable due to the randomness of the weight setting in the neural network. (4) It needs to assume that the feature vector and the target are linearly separable when using the Logistic Regression model. (5) The Random Forest and Rotation Forest algorithms are not affected by the non-linear relationship of the data and can get relatively good results. However, the selection of feature attributes of the constructed tree is random, and it will affect the prediction result when there is noise in the sample data. (6) AdaBoost and Gradient Boosting are special ensemble learning methods. In each iteration, the algorithm will update the sample weights according to the predicted effect of the trained learner and use it for a new round of learning. Different from AdaBoost, Gradient Boosting uses a spatial gradient descent algorithm to update the weights and finally achieves better results. Overall, our experiments show that Gradient Boosting has the best performance for lncRNA-disease association prediction compared with other classifiers.

### Comparison with other state-of-the-arts

We compare LDNFSGB with the following computational models: (1) *BPLLDA* [33], which is a network-based method based on simple paths with limited lengths in a heterogeneous network. (2) *IIRWR* [31], which is a random walk with restart architecture with disease clique using an internal tendency. (3) *LDASR* [38], which is an integrated machine learning method using the rotation forest. (4) *SKF*-*LDA* [39], which introduces the kernel fusion method with different types of similarities for lncRNAs and diseases. (5) *ILNCSIM* [40], which develops an improved lncRNA functional similarity calculation model based on the assumption that lncRNAs with similar biological functions tend to be involved in similar diseases. (6) *Ping et al.’s Method* [21], which constructs a bipartite network to predict potential lncRNA-disease interactions only based on the known lncRNA-disease association. (7) *Yuan et al.’s Method* [30], which is a cluster correlation based method for lncRNA-disease association prediction.

**Fig. 6:**
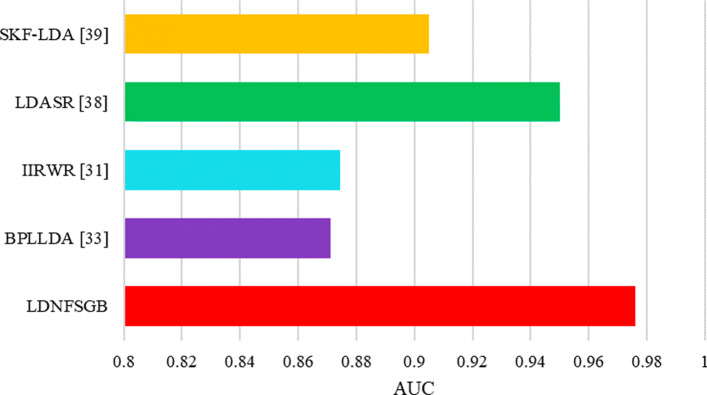
AUC comparison results of LDNFSGB with the state-of-the-art methods on LncRNADisease for lncRNA-disease association prediction

The comparison with other popular methods on LncRNADisease is shown in Fig. 6, in which, we can find that our method has the highest prediction result with an AUC of 0.9761, which improves by 2.59%-10.49%. The reasons for improvement can be attributed to two aspects. On the one hand, we comprehensively consider all the features of lncRNAs and diseases for a better representation. On the other hand, we propose a high-performance prediction model using autoencoder and gradient boosting, which are good at feature representation and integrating multiple weak learners, respectively. Comparison results have shown that LDNFSGB achieves the best performance and is of great significance for the prediction of potential lncRNA-disease associations.

Moreover, to detect the significant differences between our proposed model and other models, a t-test is used to verify the performance of LDNFSGB. Here, we find the distribution of F1-score after repeating the process twenty times. The *p*-value of the t-test between any other models vs. our method is shown in Table 5. The results demonstrate that the performance of LDNFSGB is significantly better than others in terms of the F1-score (*p*-value < 0.05).

**Table 5:** The statistical significance validation of LDNFSGB for the performance comparison using t-test

Method	Mean ± std (%)	*p*-value
LDASR [38]	88.09 ±1.054	0.01943
BPLLDA [33]	80.08 ±10.965	0.00302
LDNFSGB	88.94 ±1.067	-

**Fig. 7:**
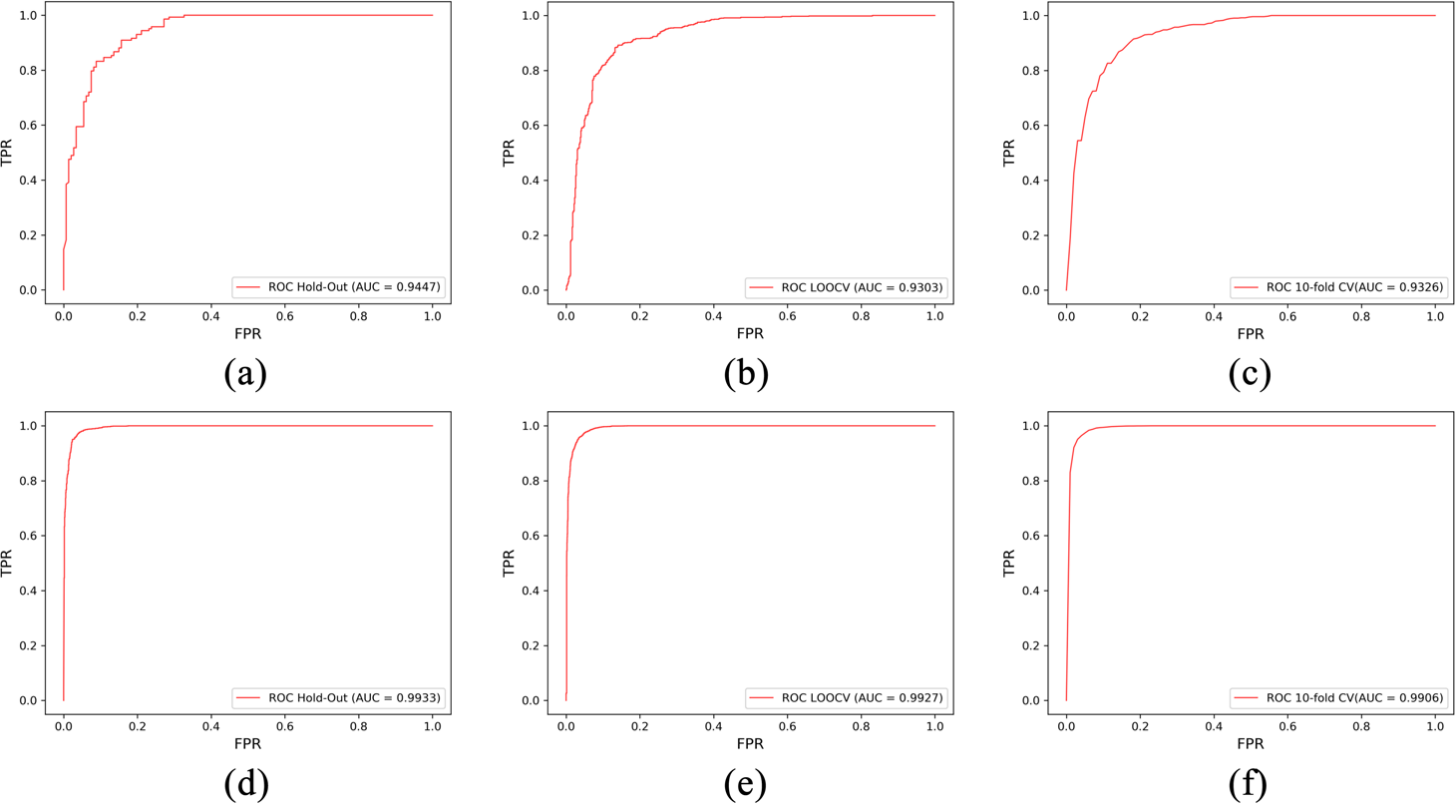
ROC curves of LDNFSGB for lncRNA-disease association prediction under hold-out, LOOCV, and 10-fold CV on Lnc2Cancer and LncRNADisease2.0. **(a)**, **(b)**, and **(c)** represent the results on Lnc2Cancer, and **(d)**, **(e)**, and **(f)** represent the results on LncRNADisease2.0, respectively

Three verification methods (i.e., hold-out, LOOCV, and 10-fold CV) are also used on Lnc2Cancer and LncRNADisease2.0 for evaluating the performance of the proposed model. The ROC curves are shown in Fig. 7. Among them, LDNFSGB using hold-out obtains the highest result with an AUC of 0.9447 and the average values of AUCs obtained by LOOCV and 10-fold CV are 0.9302 and 0.9326 on Lnc2Cancer, respectively. Furthermore, we compare the performance of our model with that of the state-of-the-art methods [21, 30, 40] on Lnc2Cancer as well. The results in Table 6 show that LDNFSGB improves the AUC by 2.09%-10.4%, which indicates that our model dramatically outperforms the competing methods. For the LncRNADisease2.0 dataset, we find that LDNFSGB achieves amazing AUC results, which are 0.9933, 0.9926, and 0.9906 using hold-out, LOOCV, and 10-fold CV respectively. Perhaps, this is mainly because LncRNADisease2.0, a larger scale dataset compared with LncRNADisease and Lnc2Cancer, has more lncRNA-disease associated pairs, and therefore, more useful information can be used for the prediction.

**Table 6:** Performance comparison of LDNFSGB and six state-of-the-art models in terms of AUC on Lnc2Cancer using LOOCV

Method	Year	AUC
LRLSLDA-ILNCSIM [40]	2016	0.9094
LRLSLDA [40]	2016	0.8263
LRLSLDA-ILNCSIM1 [40]	2016	0.9046
LRLSLDA-ILNCSIM2 [40]	2016	0.9009
Ping et al.’s Method [21]	2018	0.8983
Yuan et al.’s Method [30]	2020	0.8410
**LDNFSGB**	**-**	**0.9303**

### Cases studies

In this section, colorectal cancer, osteosarcoma, cervical cancer, and glioma are selected as cancer case studies to verify the performance of LDNFSGB in practical application. In order to ensure the integrity and authenticity of the experiment, we choose the LncRNADisease database (v2017) for model training and prediction. The CRlncRNA [41] and NCBI [42] are selected as the sources of verification results.

Colorectal cancer is the third leading cause of cancer-related deaths worldwide, with over one million new cases in Europe and the US every year [43]. It is the second most common cancer affecting women, after breast cancer, and the third most common in men, after prostate and lung cancers [25]. In this case study, the main steps are as follows: (1) After removing the samples related to colorectal cancer from the 1765 positive samples, the rest are used as positive examples, and the negative samples with the same number of positive samples are randomly selected. (2) 881 sample pairs of proven lncRNA-colorectal cancer are selected as test samples. (3) Input the training samples into LDNFSGB, and each sample outputs a probability value accordingly. (4) Sort all the results in descending order, and finally predict the lncRNA most relevant to colorectal cancer. Finally, the top 10 prediction results are verified based on existing databases and literature, as shown in Table 7. For example, overexpression of H19 decreases overall survival and increases the migration of colon cancer cells [45]. The expression of genes involved in epithelial-mesenchymal transformation is regulated by changes in SPRY4-IT1 expression. SPRY4-IT1 negatively regulates the expression of mir-101-3p in colorectal cancer cells. The results indicate that mir-101-3p binding sites may exist in SPRY4-IT1 transcripts. Therefore, SPRY4-IT1 knockout may be a reasonable treatment strategy for colorectal cancer [46].

**Table 7:** Top 10 colorectal cancer-associated lncRNAs predicted by LDNFSGB

Rank	LncRNA name	Description
1	H19	lncRNAdisease
2	SPRY4-IT1	lncRNAdisease
3	TUG1	lncRNAdisease/CRlncRNA
4	HOTTIP	lncRNAdisease/CRlncRNA
5	TCL6	unknown
6	HAR1B	unknown
7	BDNF-AS	literature [44]
8	HOTAIR	lncRNAdisease/CRlncRNA
9	ATB	CRlncRNA
10	HARLA	unknown

Osteosarcoma is a highly invasive common primary bone malignant tumor with an annual incidence of approximately (1-3) per 1,000 worldwide [12]. All experimental steps are the same as that on colorectal cancer. A total of 83 samples are related to osteosarcoma, so the number of positive samples is 1628. Similarly, 881 out of 1628 test samples are randomly selected. The validated top 10 lncRNAs are illustrated in Table 8. For example, MALAT1 increases stem cell-like properties by up-regulating RET in sponge mir-129-5p, thus activating the PI3K-Akt signaling pathway and providing potential therapeutic targets for osteosarcoma treatment [48]. CCAT1 is upregulated in osteosarcoma tissues and cells and participates in the proliferation and migration of osteosarcoma by regulating mir-148a/phosphatidylinositol 3-kinase interaction protein 1 (pik3ip1) signal pathway [49].

**Table 8:** Top 10 osteosarcoma-related lncRNAs predicted by LDNFSGB

Rank	LncRNA name	Description
1	MALAT1	lncRNAdisease
2	LINC-ROR	unknown
3	HOTAIR	lncRNAdisease
4	TUG1	lncRNAdisease/CRlncRNA
5	MIR17HG	literature [47]
6	HULC	lncRNADisease
7	BANCR	lncRNADisease
8	CCAT1	lncRNAdisease/CRlncRNA
9	BCTRN1	unknown
10	CDKN2B-AS1	lncRNADisease

Cervical cancer is currently one of the serious and high mortality cancers in the world. 200,000 of the approximately 500,000 newly diagnosed cases worldwide die from cervical cancer every year [50]. Without early diagnosis, cervical cancer develops into invasive cancer in many patients, which leads to a low survival rate. The common treatment of advanced cervical cancer is radiotherapy and nuclear chemotherapy. However, these methods are not effective and can lead to serious negative effects. To our best knowledge, lncRNA is a molecular regulatory factor in cancer, and it can provide a therapeutic target. Therefore, lncRNA research is helpful to improve the survival rate of cervical cancer patients [50]. As shown in Table 9, we predict the ten lncRNAs most related to the certificate cancer using the proposed LDNFSGB. Specifically, TUG1 can reverse the inhibitory effect of mir-138-5p on cervical cancer cells. The upregulation of TUG1 expression is closely related to the late clinical features and poor overall survival rate [51]. Besides, the overexpression of BCAR4 may be an independent prognostic factor of cervical cancer, and it can promote the proliferation and movement of cervical cancer cells [52].

**Table 9:** Top 10 cervical cancer-related lncRNAs predicted by LDNFSGB

Rank	LncRNA name	Description
1	TUG1	literature [51]
2	BACR4	literature [52]
3	GAS5	lncRNAdisease
4	H19	lncRNAdisease
5	CDKN2B-AS1	lncRNAdisease
6	MEG3	lncRNAdisease
7	HOTAIRM1	unknown
8	SPRY4-IT1	lncRNAdisease
9	HULC	lncRNAdisease
10	HNF1A-AS1	literature [53]

Glioma is the most common and aggressive malignant tumor of the central nervous system [54]. Although various treatments such as radiotherapy and chemotherapy are available, the overall survival rate for most glioma patients remains low [55]. In particular, in the case of glioblastoma, glioma patients survive only about 14 months [56]. Increased or decreased lncRNA expression can lead to tumor inhibition or promoter action. The study of glioma-related lncRNAs can provide a new direction for the diagnosis and treatment of glioma. Hence, we apply our method to predict possible lncRNAs associated with glioma. As illustrated in Table 10, nine of the top 10 predictions are proven to be related to glioma. The results indicate that overexpression of HOTTIP inhibits the growth of glioma cell lines (u87-mg, u118-mg, U251, and A172), so high levels of HOTTIP reduce glioma cell growth [57]. H19 is specifically upregulated in glioma cell lines and promotes glioma cell growth by targeting mir-140. Besides, H19-induced glioma cell growth requires mir-140-dependent P53 apoptosis-stimulating protein inhibitors (iASPP). Therefore, H19 may modulate tumor growth through MIP-140-dependent iASPP [58].

**Table 10:** Top 10 glioma-related lncRNAs predicted by LDNFSGB

Rank	LncRNA name	Description
1	HOTTIP	lncRNAdisease
2	LINC01158	lncRNAdisease
3	H19	lncRNAdisease
4	SPRY4-IT1	lncRNAdisease
5	ATB	lncRNAdisease
6	MIR100HG	unknown
7	GAS5	lncRNAdisease
8	CCAT1	lncRNAdisease
9	BCYRN1	lncRNAdisease
10	MDC1-AS1	lncRNAdisease

**Fig. 8:**
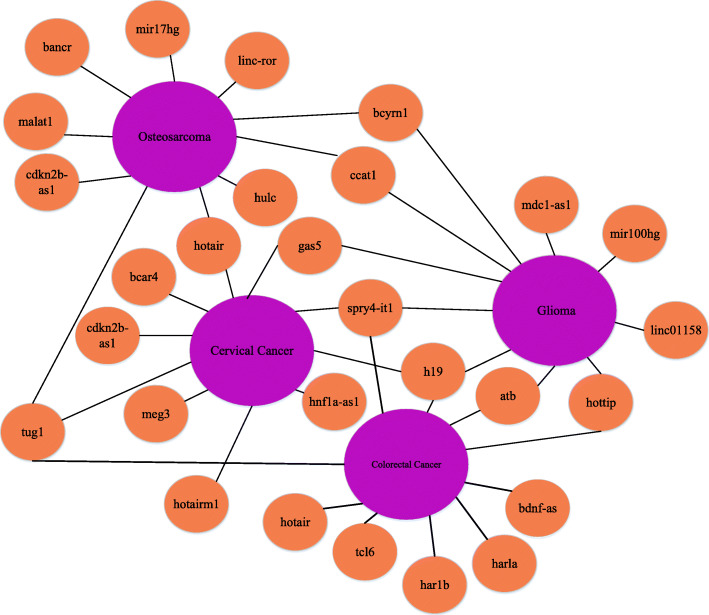
Case study of colorectal cancer, osteosarcoma, cervical cancer, and glioma. Orange nodes represent lncRNAs and purple nodes represent diseases. The top 10 scored candidate lncRNAs for each disease are linked by black edges

The visualization results of the case studies are shown in Fig. 8. If the association between lncRNA and disease is correctly predicted, it will provide a new perspective on the diagnosis and treatment of diseases. In this section, we analyze the top ten lncRNAs related to the disease and obtain 70%, 80%, 90%, and 90% accuracy, respectively. Due to the small number of samples, the current results are better than those of most existing literature. According to the above description, we can see that LDNFSGB has achieved positive and satisfactory performance in predicting potential lncRNA-related diseases.

To further verify the performance of our model for the prediction of lncRNA-disease association, heart failure and Alzheimer’s disease are selected as non-cancer case studies. Heart failure, a life-threatening condition, has been the focus of extensive research due to its ischemic, hypertensive, infectious, or hereditary nature [59]. However, evidence suggests that lncRNA has made significant advances in the understanding of gene recombination and the regulatory role of heart growth and development during heart failure [60]. It may provide an exciting opportunity for the effective treatment of heart failure. AD is a common neurodegenerative disease. An estimated five million new cases of AD are diagnosed globally each year [61]. Therefore, it is of special significance to study the regulation mechanism of lncRNA in the process of AD. With the same experimental steps as the previous four cancer-related diseases, we predict the top ten lncRNAs related to heart failure and AD, respectively. More details are presented in Table 11. Evidence shows that six of the top ten lncRNAs associated with heart failure and Alzheimer’s disease are confirmed.

**Table 11:** The lncRNAs in the top 10 for the two non-cancer case studies predicted by LDNFSGB

Disease	LncRNA name	Description	Rank
Heart failure	HULC	unknown	1
Heart failure	91H	unknown	2
Heart failure	XIST	literature [62]	3
Heart failure	TUG1	literature [63]	4
Heart failure	MEG3	literature [64]	5
Heart failure	H19	literature [65]	6
Heart failure	UCA1	literature [66]	7
Heart failure	GAS5	literature [67]	8
Heart failure	DLEU1	unknown	9
Heart failure	TP73-AS1	unknown	10
Alzheimer’s disease	91H	unknown	1
Alzheimer’s disease	HOTAIRM1	lncRNAdisease2.0	2
Alzheimer’s disease	MEG3	literature [68]	3
Alzheimer’s disease	SPRY1-IT1	unknown	4
Alzheimer’s disease	BANCR	unknown	5
Alzheimer’s disease	BCAR4	lncRNADisease	6
Alzheimer’s disease	GAS5	unknown	7
Alzheimer’s disease	H19	unknown	8
Alzheimer’s disease	NEAT1	literature [69]	9
Alzheimer’s disease	HOTAIR	literature [70]	10

Although LDNFSGB achieves satisfactory and reliable prediction performance in the prediction of potential lncRNA-disease associations, some new interesting related lncRNAs, such as DLEU1, 91H, TP73-AS1 are also undiscovered. The molecular mechanism of these related lncRNAs is still unveiled, but a new perspective is provided to validate by biological experiments for researchers.

## Discussion

Many studies have shown that machine learning-based approaches play an increasing role in lncRNA-disease association prediction, which can greatly help researchers understand complex human diseases at the biomolecular level and further provide new perspectives for diagnosis and targeted treatment. In this paper, we propose a novel method to predict potential associations between lncRNAs and diseases by using network feature similarity and gradient boosting. Firstly, a feature vector is constructed by assembling the DISS and LNCS. Especially, DISS is constructed by combining GISDS and DISSS. We use two methods to calculate DISSS, where DISSS1 considers local information on disease semantics and DISSS2 reflects global information on disease semantics. LNCS is constructed by integrating LNCGS and LNCFS. Similarly, LNCFS is also obtained using two methods to consider both the local and global information of lncRNA functions. Besides, the introduction of the Gaussian interaction profile kernel takes into account the sparsity of the lncRNA-disease interactions. Secondly, an autoencoder is used to reduce the dimensionality of the feature vector to get the optimal feature parameter from the original feature set. Thirdly, we propose to use the gradient boosting on the optical feature parameters to obtain the lncRNA-disease prediction results. In particular, the integration of the autoencoder and gradient boosting effectively reduces the complexity and training time of LDNFSGB. Finally, we evaluate our method on the LncRNADisease database (v2017) from different perspectives, e.g., different features and different classifiers using hold-out, LOOCV, and 10-fold CV, respectively. Moreover, another two datasets, i.e, Lnc2Cancer and LncRNADisease2.0 are further used to verify the performance of LDNFSGB. We also compare LDNFSGB with several state-of-the-art methods. The results have demonstrated that LDNFSGB dramatically outperforms other competing methods in terms of best AUC values. In addition, case studies have verified the effectiveness of LDNFSGB in predicting the potential associations between lncRNAs and diseases.

Although the proposed model overcomes some existing problems, it still has some limitations and there are some questions remain to be explored. For example, we only considered the functional information of lncRNA in feature extraction. However, many other characteristics of lncRNA are also very helpful for the prediction of lncRNA-disease association, such as lncRNA sequence, structure, location information, etc. In this study, we used a supervised approach to predict potential lncRNAs associated with diseases. We summarize unlabelled samples as negative samples, but unlabelled lncRNA-disease association pairs may be relevant. Therefore, unsupervised learning is expected to be a new way to further improve the performance by incorporating more useful information.

## Conclusion

In this study, we propose a novel and effective method for predicting potential lncRNA-disease associations using network feature similarity and gradient boosting. We first construct a comprehensive feature vector to extract the global and local information of lncRNAs and diseases. Then, an autoencoder is employed to reduce the dimensionality of the feature vector to obtain the optimal feature parameter from the original feature set. Furthermore, we utilize the gradient boosting algorithm to obtain the lncRNA-disease association prediction. Finally, we evaluate the proposed method on three publicly available datasets. Moreover, we also compare our method with several state-of-the-art approaches. The results and case studies have demonstrated the effectiveness of our method in predicting lncRNA-disease associations.

## Methods

### Datasets

The first dataset used in this paper is LncRNADisease (v2017) [71], and the known lncRNA-disease association data was downloaded from the LncRNADisease database. After eliminating duplicate descriptions of lncRNA-disease associations and invalid samples, we obtain 1765 lncRNA-disease related sample pairs and 287,203 lncRNA-disease uncorrelated sample pairs, including 881 lncRNAs and 328 diseases. We summarize these 1765 lncRNA-disease association candidates as positive samples. To eliminate the imbalance problem of samples, we randomly select 1765 out of 287,203 unassociated candidates as the final negative samples.

To comprehensively evaluate the performance of LDNFSGB, another two datasets, i.e., Lnc2Cancer [72] and LncRNADisease2.0 [73] are used. Lnc2Cancer, a manually managed dataset, provides experimentally supported associations between lncRNAs and cancers by consulting more than 6,500 published papers. A dataset consisted of 725 known lncRNA-disease associations can be obtained using the same pre-processing as the LncRNADisease dataset, which includes 355 lncRNAs and 76 diseases. LncRNADisease2.0 is an updated version of the LncRNADisease dataset, which adds a lot of new lncRNA and disease associations. Similarly, we get 7981 known lncRNA-disease associations including 6076 lncRNAs and 452 diseases.

The disease semantic similarity data were retrieved from the Medical Subject Heading (MeSH). The MeSH, which is a definitive subject vocabulary compiled by the National Library of Medicine, provides hierarchical organizational terms for indexing and classifying various diseases. It is the source for constructing directed acyclic graphs (DAGs) [74].

#### Construction of the lncRNA-disease interaction matrix

The known lncRNA-disease interaction is the basis for calculating the similarity of all features and is also the label of the model. After quantifying the lncRNA-disease related sample pairs, an adjacency matrix is constructed based on the known lncRNA-disease interaction and called LNCDIS. The matrix $LNCDIS=[M_{ij}^{LNCDIS}]\in R^{N_{d} \times N_{l}}$ represents the association pairs between *N*_*l*_ lncRNAs and *N*_*d*_ diseases, where $M_{ij}^{LNCDIS}$ is 1 if disease *d*_*i*_ is associated with lncRNA *l*_*j*_. Otherwise, $M_{ij}^{LNCDIS}$ is 0.

### Similarity measures

#### Construction of the disease semantic similarity matrix

There are currently two methods for calculating the semantic similarity of diseases, which are named DISSS1 and DISSS2, respectively. DISSS1, which only takes into account the local information on disease semantics, thinks the more related to the semantics of diseases, the greater the contribution of diseases. However, DISSS2 believes that the higher the frequency of diseases, the greater the contribution of diseases [74], and it takes into account global information on disease semantics. Taking both ideas of DISSS1 and DISSS2 into consideration, we herein employ two similarity calculation methods to obtain the disease semantic similarity matrix. The calculation of DISSS1 is mainly as follows.

**Fig. 9:**
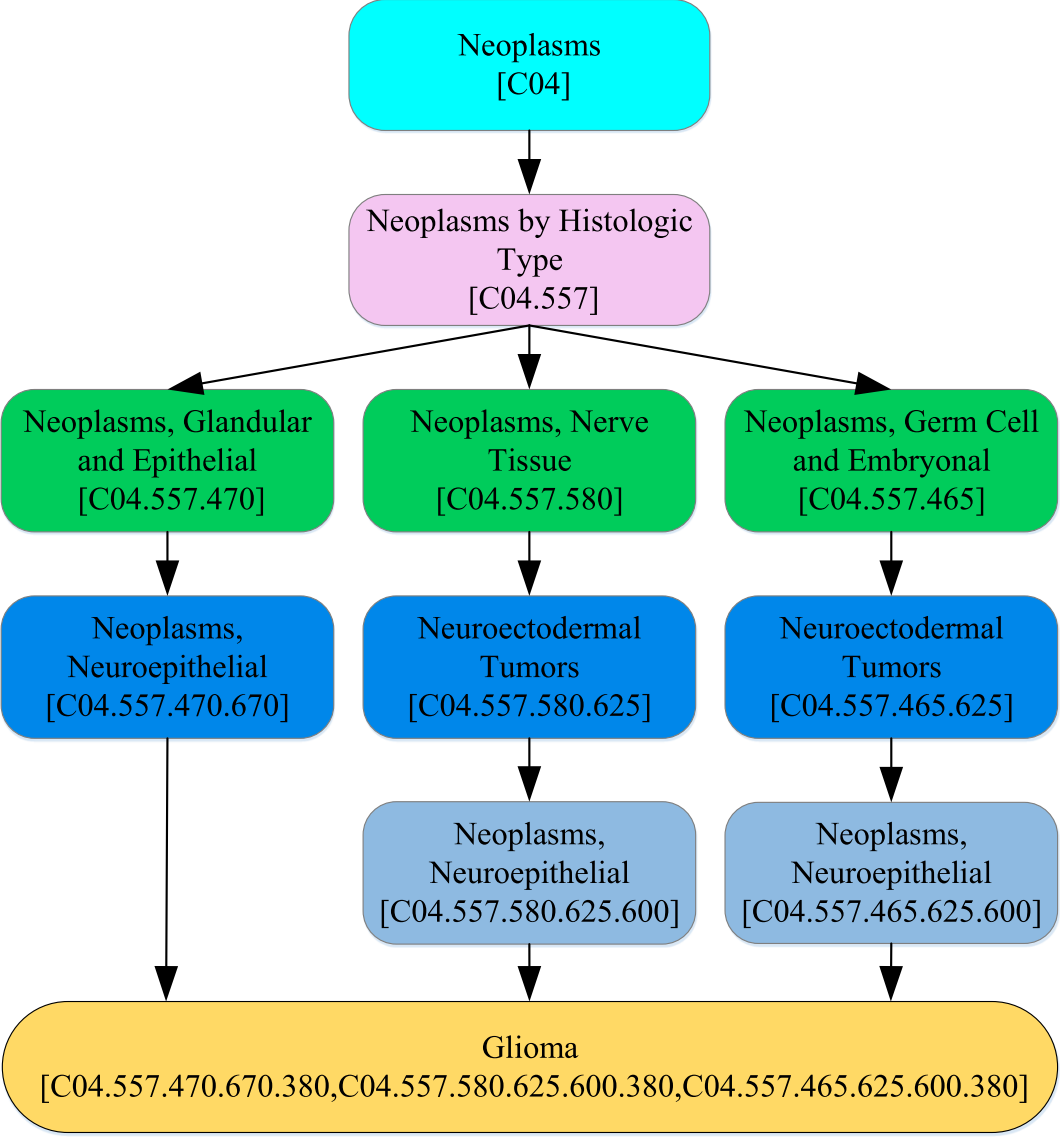
The DAG of the glioma

(1) We download the MeSH descriptions of diseases from the National Medical Library of Medicine. These descriptions provide detailed semantic information for each disease.

(2) Based on the obtained MeSH information, we construct a direct graph for each disease. The DAG of the glioma is shown in Fig. 9.

(3) DAGs are used to calculate the disease semantic similarity. A disease *d* can be described as *DAG*(*d*)=(*d*,*D*(*d*)), where *D*(*d*) is the node-set of *d* and all of its ancestor nodes. For any disease *k*∈*D*(*d*) in DAG, its semantic contribution to *d* is defined as [75]

6 $$ \left\{\! {\begin{array}{*{10}{l}} {DS{1_{d}}(k) \! =\! 1}& {if{ \ }k = d}\\ \! {\ DS{1_{d}}(k)\! =\! max \left\{\delta \! \times \! {DS1\ }_{d}(k^{\prime})|k^{\prime} \in D(d) \right\}} \! & \! {otherwise} \end{array}} \right.  $$

where *δ* represents the semantic contribution decay factor for the edge among disease nodes. It is specified by 0<*δ*<1 and is generally set as 0.5.

(4) We calculate the final contribution of disease *d* as follows:

7 $$ D1(d)=\sum_{k\in D(d)}{{DS1}_{d}(k)}  $$

(5) Then, the association between the two diseases can be calculated by

8 $$ DISSS1\left(i,j\right)=\frac{\sum_{k\in D(i)\cap D(j)}{({D1}_{i}(k)+{D1}_{j}(k))}}{||D1(i)||+||D1(j)||}  $$

For DISSS2, the pipeline is as follows:

(1) The semantic contribution to disease *d* is defined as

9 $$ { DS2}_{D}(d)=\log{\left(\frac{||DAG(d)||}{N_{d}}\right)}  $$

(2) The final semantic value of disease *d* can be calculated by

10 $$ D2(d)=\sum_{k\in D(d)}{{DS2}_{d}(k)}  $$

(3) Therefore, the association between the two diseases can be calculated by

11 $$ DISSS2\left(i,j\right)=\frac{\sum_{k\in D(i)\cap D(j)}{({D2}_{i}(k)+{D2}_{j}(k))}}{||D2(i)||+||D2(j)||}  $$

Finally, we can obtain disease semantic similarity matrices $DISSS1=[M_{ij}^{DISSS1}]\in R^{N_{d} \times N_{d}}$ and $DISSS2=[M_{ij}^{DISSS2}] \in R^{N_{d}\times N_{d}}$ respectively, where both *DISSS*1_*ij*_ and *DISSS*2_*ij*_ denote the similarity values between *D*(*i*) and *D*(*j*). *N*_*d*_ is the number of diseases.

#### Construction of the lncRNA function similarity matrix

After obtaining the feature vector of semantic similarity of diseases, we adopt a similarity method proposed by Chen et al.[20] to calculate the functional similarity of lncRNAs. Supposing lncRNA *p* is related with a disease set *D*_*p*_={*d*_*k*_|1≤*k*≤*m*} and lncRNA *q* is associated with a disease set *D*_*q*_={*d*_*l*_|1≤*l*≤*n*}. Especially, *m* is the total number of diseases related to lncRNA *p* and *n* is the total number of diseases related to lncRNA *q*. The degree of association between lncRNA *p* and disease *D*_*q*_ is

12 $$ LS1\left(p,D_{q}\right)=max\left\{DISSS1\left(d_{k},d_{l}\right)\right\}  $$

The functional similarity between *p* and *q* is calculated as

13 $$ \begin{array}{rcl} LNCFS{1_{p,q}} = \frac{{{\sum\nolimits}_{1 \le k \le m} {LS1({d_{k}},{D_{q}})} }}{{m + n}} + \frac{{{\sum\nolimits}_{1 \le l \le n} {LS1({d_{l}},{D_{p}})} }}{{m + n}} \end{array}  $$

We can obtain the lncRNA function similarity matrix $LNCFS1=[M_{ij}^{LNCFS1}] \in R^{N_{l} \times N_{l}}$. Similarity, we can also get $LNCFS2=[M_{ij}^{LNCFS2}] \in R^{N_{l} \times N_{l}}$, where *N*_*l*_ denotes the number of lncRNAs. Obviously, *LNCFS*1 and *LNCFS*2 are symmetric matrices.

### LDNFSGB

#### Methods overview

**Fig. 10:**
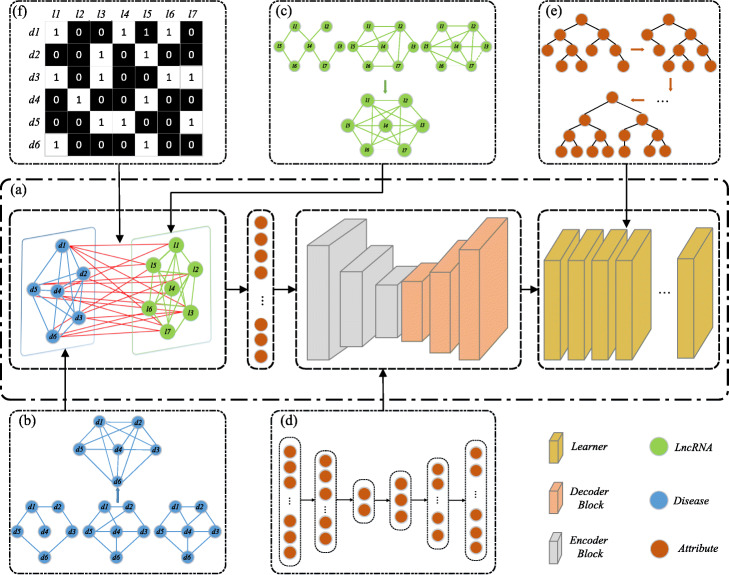
The flowchart of the proposed LDNFSGB. **(a)** The main workflow of LDNFSGB. **(b)** The construction process of DISS. **(c)** The construction process of LNCS. **(d)** Autoencoder. **(e)** The gradient boosting algorithm. **(f)** LNCDIS

In this paper, we propose LDNFSGB to predict lncRNA-related diseases. The main workflow of LDNFSGB is illustrated in Fig. 10a. We firstly construct a comprehensive feature vector to effectively extract the global and local information of diseases and lncRNAs by combining DISS and LNCS. As shown in Fig. 10b, the average of DISSS1 and DISSS2 is taken for the disease semantic similarity network. Then, we get DISS by combining DISSS and DISGC. As shown in Fig. 10c, the average of LNCFS1 and LNCFS2 is taken for the lncRNA function similarity network. Similarly, the LNCS is obtained by combining LNCFS and LNCGS as well. Secondly, we utilize an autoencoder to reduce the dimensionality of the feature vector to get the optimal feature parameter from the origin feature set (Fig. 10d). Finally, more discriminative feature vectors are put into the gradient boosting for training, testing, and prediction based on the regression tree (Fig. 10e). Besides, Fig. 10f shows the association between known lncRNAs and diseases. It is the basis for all feature calculations and the label of the model.

#### Construction of the Gaussian interaction profile kernel similarity matrix for lncRNAs and diseases

To eliminate the effects caused by missing MeSH information and lots of zero values in the lncRNA-disease adjacency matrix, the Radial Basis Function (RBF) Gaussian kernel function is utilized. Given diseases *D*(*i*) and *D*(*j*), the Gaussian interaction profile kernel similarity between them can be represented as

14 $$ DISGS\left(i,j\right)=e^{(-\mu_{d}||D(i)-D(j){||})^{2}}  $$

where *μ*_*d*_ is a weight used to control the bandwidth of the kernel, which can be calculated by

15 $$ \mu_{d}=\mu_{d}^{\prime}\left(\frac{1}{N_{d}}\sum_{i=1}^{N_{d}}{||D(i){||}^{2}}\right)  $$

where *N*_*d*_ represents the number of diseases and the best value of $\mu _{d}^{\prime }$ is 0.5. Obviously, the third disease semantic similarity matrix $DISGS=[M_{ij}^{DISGS}]\in R^{N_{d} \times N_{d}}$ is symmetric. Similarly, we can obtain the third lncRNA function similarity matrix $LNCGS=[M_{ij}^{LNCGS}]\in R^{N_{l} \times N_{l}}$, where *N*_*l*_ is the number of lncRNAs.

#### Construction of feature vector

The integration is performed to obtain the final disease semantic similarity feature vector based on the *DISSS*1, *DISSS*2, and *DISGS*.

16 $$ DISS = \left\{ \begin{array}{*{20}{l}} DISSS_{i,j} & \! \!{{if \ {\rm{ DISSS}}{1_{ij}} \cup DISSS{2_{i}}_{j} = 1}}\\ DISGS_{i,j} & \! \! {otherwise} \end{array} \right.  $$

where

17 $$ DISSS_{i,j}= \frac{{{\rm{DISSS}}1_{i,j} + DISSS2_{i,j}}}{{\rm{2}}}  $$

Similarly, we can obtain the lncRNA functional similarity feature vector based on the *LNCFS*1, *LNCFS*2, and *LNCGS*, which is called as *LNCS*. Remarkably, all similarity matrices are symmetric.

#### Autoencoder

After feature extraction, dimensionality reduction is necessary to increase the performance and efficiency of classifiers. Here, the autoencoder is chosen to obtain the discriminative feature subsets. In general, autoencoder is mainly composed of an encoder and a decoder. The encoder is used to reduce the dimensionality of the input data and the decoder contributes to restoring initial input data. The vital steps are presented as follows:

(1) Assuming the activation functions of the encoder and decoder are defined as *h*(*x*) and *g*(*k*) respectively, and both of them can be represented using a Sigmoid function by

18 $$ h(x)=\frac{1}{1+e^{-(wx+b)}}  $$

19 $$ g(k)=\frac{1}{1+e^{-(\beta k+\gamma)}}  $$

where *w* and *β* are the weights of the encoder and decoder, *b* and *γ* are the thresholds of the encoder and decoder, respectively.

(2) We employ a loss function to represent the difference between the original input and the prediction, which is defined as

20 $$ Loss\,=\, -\!\!\! {\sum\nolimits}_{i = 1}^{n} \!\! {[{x_{i}} \! \log \!(g\!(f({x_{i}}\!)\!)\!) + }\!(1 - {x_{i}}\!)\log\!(1 - g\!(f\!({x_{i}}\!)\!)\!)]   $$

where the Loss function is based on logistic regression, *g*(*f*(*x*_*i*_)) represents the feature value after encoding and decoding. *x*_*i*_ represents the original input feature value.

Finally, the optimal and dimensionality-reduced feature vector *X* is obtained based on Eq. (20) through multiple iterations.

#### Gradient boosting

In this paper, we employ a gradient boosting algorithm as the classifier for the prediction of lncRNA-disease associations. Gradient boosting is an ensemble model that uses a regression tree as a basic learner [76]. In this model, the main parameters are the maximum tree depth *d*, the number of regression tree *q*, and the learning rate *η*.

Supposing $X={[{X_{1}},{X_{2}},{X_{3}},\ldots,{X_{{N_{d}}}}]^{T}}\phantom {\dot {i}\!}$ is the optimal and dimensionality-reduced feature vector and $Y = [{y_{1}},{y_{2}},{y_{3}},\ldots,{y_{{N_{d}}}}]\phantom {\dot {i}\!}$ is the label of sample pairs. The predicted value of each weak learner ${\hat {y}}_{i}$ can be obtained by

21 $$ \widehat {{y_{i}}} = - {\left[ {\frac{{\partial L({y_{i}},{F_{m}}({X_{i}}))}}{{\partial {F_{m - 1}}({X_{i}})}}} \right]_{i = 1,2,3,...,{N_{d}}}}  $$

where *F*_*m*_(*X*_*i*_) is a function of the weak learner.

Each learner is obtained by fitting the gradient descent algorithm based on the error of the previous function as

22 $$ {F_{m}}(x) = {F_{m - 1}}(x) + {\rho_{m}}{h_{m}}(x)  $$

where

23 $$ \ h_{m}(X_{i})=-\frac{1}{2}\frac{\partial}{\partial f_{m-1}\left(X_{i}\right)}{(y_{i},F_{m-1}\left(X_{i}\right))}^{2}  $$

The goal of *h*_*m*_(*X*_*i*_) is to find the direction of the spatial gradient descent of *f*_*m*−1_(*X*_*i*_), so that the error propagates faster. *ρ*_*m*_ is defined as

24 $$ {\rho_{m}} = \mathop {\arg \min }\limits_{\rho} \sum\limits_{i = 1}^{N} {L({y_{i}},{F_{m}}({X_{i}}) + \rho {h_{m}}({X_{i}};{w^{*}}))}  $$

where *ρ* represents the search step size when finding the direction of the fastest gradient descent based on the line search method. *L*(*y*_*i*_,*F*_*m*−1_(*x*)+*ρ*_*m*_*h*_*m*_(*x*)) is the mean square error loss function. *w*^∗^ is the weight of *F*_*m*−1_(*X*_*i*_), which is define as

25 $$ {w^{*}} = \mathop {\arg \min }\limits_{w} \sum\limits_{i}^{{N_{d}}} {{{(\widehat {{y_{i}}} - {h_{m}}({X_{i}};{y_{i}}))}^{2}}}  $$

Gradient boosting is an ensemble learning algorithm. The specialty of this algorithm is that it directly updates the parameters based on the model functions. Therefore, it can extend the additivity of parameters to function space. For example, in the *m*-*th* iteration of the model, a new learner *f*_*m*_ is firstly obtained using the previous *m*-1 base learners (*f*_0_ - *f*_*m*−1_), and then *ρ*_*m*_ and *w*^∗^ can be updated continuously in the direction of gradient descent. The procedure of gradient boosting is summarized in Algorithm 1.


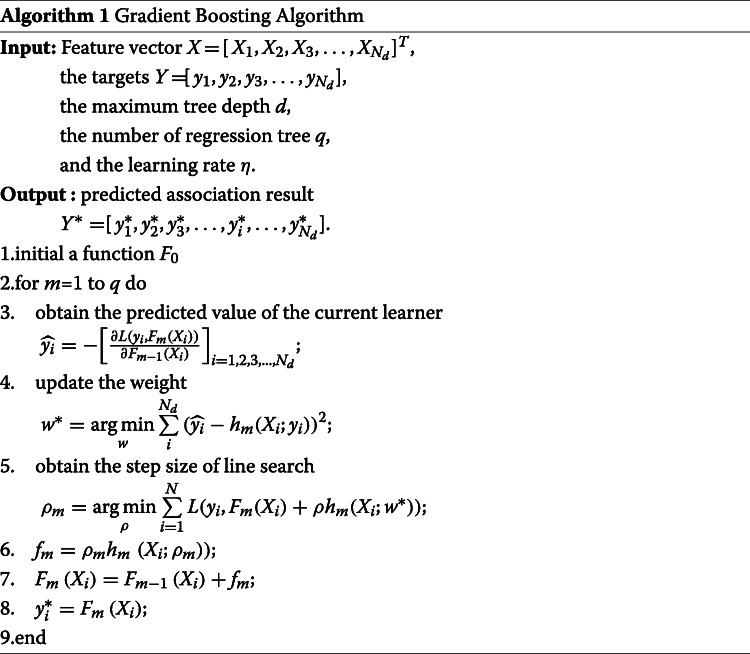


## Data Availability

The code of LDNFSGB and data processing are available at https://github.com/MLMIP/LDNFSGB.

## Abbreviations

ncRNAs: Non-coding RNAs
lncRNAs: Long non-coding RNAs
LOOCV: Leave-one-out cross-validation
ROC: Receiver operating characteristic
AUC: Area under ROC
FPR: False positive rates
TPR: True positive rates
Acc: Accuracy
Sen: Sensitivity
Spe: Specificity
Pre: Precision
MCC: Matthews correlation coefficient
MeSH: Medical subject heading
DAGs: Directed acyclic graphs.
